# Supplementary material for: Are carbon emissions trading and green financial instruments synergistic? -Comprehensive quantitative research based on content analysis
Source: PLoS One. 2024 Mar 7;19(3):e0298601. doi: 10.1371/journal.pone.0298601 (PMC10919601; doi:10.1371/journal.pone.0298601)
Supplement: S1 Data — (ZIP) [file pone.0298601.s001.zip › Data/地级市Z_F工作报告.zip!/╕ú╜¿/╕ú╓▌╩╨/└┤╫╘╣1⁄2╓┌║┼í╛┬φ┐╦╩2╛▌═°í┐_2017.docx]

2017年1月10日在福州市第十五届人民代表大会第一次会议上

　　福州市人民政府代市长 尤猛军

各位代表：

　　现在，我代表福州市人民政府，向大会报告政府工作，请予审议，并请市政协各位委员提出意见。

　　一、2016年暨市十四届政府工作回顾

　　2016年，在省委、省政府和市委的正确领导下，市政府全面贯彻党的十八大和十八届三中、四中、五中、六中全会精神，深刻领会习近平总书记系列重要讲话精神，认真落实省第十次党代会、市第十一次党代会决策部署，更新工作理念，改进工作方法，以一线考察干部为契机，以解决问题为导向，以落实项目为抓手，深入开展“抓征迁、促落实”“抓招商、促发展”“抓治理、促提升”等专项行动，着力破解群众关注的热点难点问题，各项工作都取得新的进展。一年来的主要工作和成效是：

　　（一）经济增长稳中有升。预计全市地区生产总值6050亿元，增长8.3%；一般公共预算总收入934.06亿元，增长10.1%；地方一般公共预算收入598.91亿元，同口径增长11.7%；固定资产投资5170亿元，增长6.5%；出口总额1430亿元，增长9%；实际利用外资18.14亿美元，增长8.1%；社会消费品零售总额3883亿元，增长11.3%；居民消费价格总水平上涨2.5%；城镇居民人均可支配收入3.78万元，增长8%；农村居民人均可支配收入1.65万元，增长8.5%；城镇登记失业率2.43%。省下达的节能减排降碳任务全面完成。

　　（二）产业发展提质增效。服务业增加值突破3000亿元，占GDP的比重超50%。规模以上工业增加值1980亿元，增长7.8%。工业技改投资800亿元，增长1.3%。申远聚酰胺、吴航不锈钢技改等136个重点产业项目加快建设。出台供给侧结构性改革“1+5”系列文件，化解钢铁行业过剩产能50万吨，减少房地产库存26%，减轻企业负担217亿元。全国首个VR产业基地落户中国东南大数据产业园，全国首家窄带物联网规模化商用局在马尾启用。福州入选全国服务外包示范城市、全国“十三五”服务业综合改革试点区域、全国“十三五”海洋经济创新发展示范城市、国家首批健康医疗大数据中心与产业园建设试点城市、中国领军智慧城市。海峡金融商务区和闽江北岸中央商务区已落户总部经济项目44个。成功举办第95届全国糖酒会、第34届体博会等85场展会活动。

　　（三）国家战略扎实推进。加快福州新区开放开发，完成年度投资1780亿元，占年度计划的111.5%，组建新区开发投资集团，设立新区投资发展基金。全面融入海丝核心区建设，制定“海上福州”建设工作方案，启动实施10项任务、195个重点项目。推动福建自贸试验区福州片区先行先试，推出三批共46项创新举措、两批共41个金融创新案例，新增企业1.6万户。推进国家生态文明试验区建设，完成10项改革任务，工业排污企业推行排污权交易，顺利通过国家生态市考核验收。启动国家自主创新示范区福州片区建设，着手实施5大任务。

　　（四）“三农”基础不断夯实。全市农林牧渔业总产值839亿元，增长3.5%。新增全国休闲农业与乡村旅游示范县1个、国家级农业产业化重点龙头企业1个、省级农业产业化重点龙头企业50个。新建改造农村公路213公里，“一闸三线”工程、闽清葫芦门水库等69个重大水利项目加快建设。江阴、青口省级“小城市”培育试点扎实推进。建成420个新农村“幸福家园工程”示范村、129个美丽乡村。农村土地承包经营权、宅基地及集体建设用地确权登记颁证工作有序推进，土地流转面积59.44万亩。10667人实现脱贫，完成“造福工程”搬迁4683人。

　　（五）各项改革持续深化。取消和暂停110项行政审批事项、74项前置审批条件，取消65个办理环节。在全省率先全面推行企业登记“五证合一、一照一码”，在行政审批中全面推行“窗口无否决权”机制。出台市属国有企业负责人薪酬制度改革意见，“海峡环保”上市顺利推进，福州成为国务院国资委指导监督地方国资工作联系点。全面实施“营改增”，为企业减税40亿元。落地PPP项目9个，总投资174.85亿元。完成全市党政机关公务用车制度改革。全面启动不动产统一登记。

　　（六）对外开放有效拓展。建立大招商工作机制，在北京等地成功举办福州投资环境推介会，引进八仙过海旅游、福能（江阴）化学园等一批重大项目。成功举办第十八届海交会。新批千万美元以上外（台）资项目77项、境外投资项目83项。跨境电子商务公共服务平台正式运行，中国—东盟海产品交易所设立马来西亚分中心，江阴整车进口8311辆。成功举办第四届海峡青年节、第九届海峡两岸合唱节、2016海峡两岸民俗文化节等重大交流活动，台湾银行福州分行在榕开业，台商投资区、台湾农民创业园等加快建设。成功举办首届联合国海陆丝绸之路城市联盟论坛、第二届海上丝绸之路国际旅游节、第三届丝绸之路国际电影节等重大活动，建成海上丝绸之路展示馆。

　　（七）城市建设管理水平稳步提高。完成全市“多规合一”工作。建成晋安南路等57条市政道路，绕城高速东南段、长平高速等9个重点快速通道项目建设进展顺利。地铁1号线全线试运营，2号线全线动建，6号线启动建设。新辟优化公交线路88条，新增更新新能源公交车594辆，新增公共停车泊位7509个，新投放便民自行车2055辆。三江口、鹤林两个海绵城市试点片区启动建设，建成琅岐环岛路西北段等地下综合管廊主体结构4.5公里。实施旧屋区改造692万平方米，拆除“两违”692.16万平方米。基本建成保障性安居工程3.3万套，五城区完成回迁安置1.71万户、239.16万平方米。城市空气质量优良率98.6%，综合指数位居全国74个重点城市第5位。新型智慧城市标杆市启动建设。

　　（八）百日攻坚专项行动深入开展。针对项目落地难问题，启动百日攻坚专项行动，完成拆迁578.39万平方米，完成交地3.66万亩，解决问题1562个，提速项目223个，开工项目139个，完成投资1473.47亿元。围绕群众关注的热点难点问题，启动实施城区内涝治理项目71个、内河水体黑臭治理项目194个、内河沿岸污染源整治项目66个、水系周边环境整治项目39个、城区交通拥堵治理项目252个、南北火车站综合整治项目228个、全民绿化项目534个、休闲步道项目12个。城区47个易涝点建立责任人制度。新改扩建江心公园、南公园等10座城市公园。完成造林绿化12.16万亩。

　　（九）社会事业协调发展。完成63件为民办实事项目。在全省率先成立职业院校联盟，增设19个中职专业，5所普通高中达标晋级，数字青少年宫等22个项目入选省中小学德育建设示范项目。基本完成103个文化站建设和1602个文化服务中心达标提升工程，成功举办第五届闽都文化论坛暨闽都文化艺术巡回展。实施城乡居民基本医保一体化政策，初步建立分级诊疗制度，组建福州儿科医疗联合体，有效防控登革热疫情，全面两孩政策平稳实施。成功举办第24届市运会、中国羽毛球公开赛、环福州·永泰国际公路自行车赛、福州国际马拉松赛等大型体育赛事，实施590个全民健身工程，开展32项1080场全民健身活动。建立重大安全隐患点责任人制度，安全生产形势持续保持平稳。全力抗击“尼伯特”“莫兰蒂”等超强台风，有序有效开展灾后恢复重建，闽清、永泰227项灾后重建项目按计划推进。

　　（十）政府自身建设切实加强。深入开展“两学一做”学习教育，持续纠正“四风”问题。认真贯彻市委的决策部署，马上就办、真抓实干，抓好各项工作落实，初步构建上下贯通、横向联动的督查工作机制。坚决整治“为官不为”“庸懒散拖”等问题，实施效能问责221人次。行政监察、审计监督力度加大，反腐倡廉深入推进。认真执行市人大及其常委会决定决议，自觉接受市人大及其常委会的法律监督、工作监督和市政协的民主监督，积极支持市政协开展民主协商。提请市人大常委会审议地方性法规草案2件，制定政府规章和规范性文件37件。办复441件省、市人大代表建议和446件省、市政协提案，满意率分别为98.4%、99.8%。

　　2016年各项任务的完成，为市十四届政府的工作画上了圆满的句号。回顾过去的五年，全市上下抢抓机遇、奋力拼搏，团结一心、砥砺前行，谱写了福州科学发展、跨越发展的新篇章。

　　过去的五年，是福州产业集聚壮大、综合实力稳步提升的五年。地区生产总值突破6000亿元，年均增长10.3%；人均地区生产总值突破1.2万美元，年均增长9.2%；一般公共预算总收入突破900亿元，年均增长13%。形成了纺织化纤、轻工食品、机械制造、冶金建材、电子信息等5个千亿元产业，培育了捷联电子、金纶高纤等12家百亿元企业。福清、长乐、闽侯进入全国综合实力百强县，鼓楼区进入全国综合实力百强区。福州成为全国十大“互联网+”标杆城市、全国首批创建国家电子商务示范城市，蝉联全国文明城市、全国双拥模范城、国家卫生城市、全国科技进步先进市、国家知识产权示范城市等称号。

　　过去的五年，是福州改革开放不断深化、发展活力持续增强的五年。开展五轮简政放权，共取消、下放审批事项528项，市行政服务中心、市市民服务中心、市公共资源交易服务中心建成运行。国资监管和国企改革稳步推进，实施市属国企布局调整和资产整合重组，出台国资监管责任清单和履职服务清单，38家国企实现政企脱钩。社会治理、农业农村、财政金融、投融资及社会事业领域改革取得新进展。开放型经济快速发展，累计实际利用外资72.72亿美元，来榕投资或设立机构的世界500强企业新增11家。榕台合作不断深化，累计批准台资项目317项、实际利用台资3.44亿美元。对外交流更加紧密，福州成为联合国海陆丝绸之路城市联盟创始成员城市，新增国际友城和友好交流城市5个。榕港榕澳合作、省内省外协作、对口帮扶等工作扎实开展。

　　过去的五年，是福州城市框架逐步拉开、城乡统筹发展扎实推进的五年。建成区面积从232.12平方公里扩大到265.33平方公里，常住人口城镇化率从63.3%提高到68.3%。基础设施日趋完善，福州航空公司成立并投入运营，新增高速公路196公里、城市道路214公里，在全省率先实现“县县通高速、镇镇有干线、村村通客车”。宜居品质不断提高，旧屋区改造、环境综合整治、内河整治、“两违”治理等工作深入开展，建成海峡奥体中心等一批标志性建筑。大力开展国家森林城市创建活动，森林覆盖率、空气质量优良率保持在全国前列。新型城镇化、新农村“幸福家园工程”和美丽乡村建设加速推进，农村面貌明显改观。

　　过去的五年，是福州社会事业迅速发展、公共服务全面提升的五年。全社会研究与试验发展经费投入占GDP比重1.76%，每万人有效发明专利拥有量12件，均位居全省前列。各级各类教育协调发展，在全省率先实现全市县域义务教育基本均衡，在线德育、幸福教育、未成年人思想道德建设等工作走在全国、全省前列，整合组建闽江师范高等专科学校。基层卫生服务覆盖率达100%，全市公立医院改革深入推进，组建6个医疗联合体。朱紫坊、上下杭等历史文化街区（风貌区）保护开发稳步推进，三坊七巷荣获联合国亚太地区“文化遗产保护奖”并获评国家5A级景区，“福州茉莉花与茶文化系统”入选联合国全球重要农业文化遗产，获得“中国脱胎漆艺之都”称号。成功举办第一届全国青运会、2012年央视中秋晚会等重大文体活动。广播影视、哲学社会科学、科普、气象、防震、地方志、档案、老龄、妇女儿童、残疾人、慈善等各项事业全面发展。

　　过去的五年，是福州民生保障日益完善、人民幸福指数不断提高的五年。全市各级财政累计用于民生支出2372.24亿元，累计为城乡人民兴办实事项目368件。城镇居民人均可支配收入、农村居民人均可支配收入分别比2011年增长57.8%、67.2%。累计实现城镇新增就业71.99万人，转移农业富余劳动力25.15万人。累计建成保障性安居工程12.8万套，实施259个旧屋区改造项目。城乡居民基本养老保险制度实现全覆盖，城乡居民基础养老金、城乡低保和医保财政补助标准持续提高，养老服务体系初步形成，社会救助、社会福利、优抚安置等工作有效开展。精准扶贫、精准脱贫成效明显，实现国定扶贫标准下贫困人口全部脱贫。计划生育服务管理不断改进，居民健康水平明显提升。平安福州、法治福州建设不断深化，网格化社会服务管理工作全面开展，省会城市保持安定稳定。民族宗教、信访、行政复议、司法以及国防动员、双拥共建、民兵预备役建设、海防、人防、反走私等工作进一步加强。

　　各位代表，成绩来之不易，这是省委、省政府和市委坚强领导的结果，这是全市人民团结奋斗、社会各界大力支持的结果。在此，我代表市人民政府，向全市人民，向人大代表、政协委员、各民主党派、工商联、无党派人士、各人民团体、离退休老同志和社会各界人士，向中直、省直机关企事业单位和驻榕部队、武警官兵、公安政法干警，向关心支持福州发展的港澳台同胞、海外侨胞和国际友人，表示衷心的感谢！

　　在总结成绩的同时，我们也看到当前福州发展中存在的困难和问题，主要是：经济总量不够大，产业结构不够优，创新能力不够强；城乡、县域之间发展不够均衡，优质教育、医疗供给不足，社会保障水平不够高，社会诚信体系尚未健全；生态环境保护与节能减排压力较大，应对重大自然灾害能力亟待提升。此外，少数公务人员推诿扯皮、消极腐败等问题还时有发生。对此，我们将采取有效措施，认真加以解决。

　　二、新一届政府努力方向和2017年工作安排

　　各位代表，根据市第十一次党代会部署，今后五年我市经济社会发展的总体要求是：高举中国特色社会主义伟大旗帜，坚持以马克思列宁主义、毛泽东思想、邓小平理论、“三个代表”重要思想、科学发展观为指导，深入贯彻习近平总书记系列重要讲话精神和对福建、福州工作的重要指示，紧紧围绕“五位一体”总体布局和“四个全面”战略布局，自觉践行五大发展理念，积极抢抓“五区叠加”战略机遇，以务实的精神补短板，以创新的思维促改革，以赶超的勇气谋发展，“马上就办、真抓实干”，态度再坚决一点、行动再快一点，坚持高标准、严要求，加快建设“机制活、产业优、百姓富、生态美”的新福州，率先全面建成小康社会，努力当好带动全省经济社会发展的排头兵。为此，今后五年，新一届政府应朝着以下目标进行不懈努力：

　　——排头兵作用更加凸显。省会、生态、海洋、侨台等比较优势得到充分发挥，发展的平衡性、协调性、可持续性位居全省前列。福州新区建设取得重大突破，成为带动全省加快发展的新引擎。福州大都市区建设格局加快形成，带动全省区域一体化发展。先行先试、率先发展的示范作用持续增强，引领全省发展方式转变。

　　——产业支撑更加有力。全面完成“十三五”发展规划，地区生产总值年均增长9%左右，经济总量提前实现比2010年翻一番，在全国省会城市中位次前移。构建现代产业体系，产业发展迈向中高端，培育形成1个三千亿元产业、4个两千亿元产业、2个一千亿元产业和20家百亿元企业。“海上福州”建设全面推进，国际旅游城市建设取得突破，现代服务业主体作用不断增强。

　　——体制机制更加灵活。主动对接国家战略，“五区”建设取得重大进展。打造最优投资软环境，法治化、国际化、便利化的营商环境加快形成，对外开放不断拓展。重点领域和关键环节改革取得决定性成果。闽都人才集聚区建设加快推进，创新型省会城市建设取得明显成效。

　　——人民生活更加幸福。提前实现城乡居民人均可支配收入比2010年翻一番，省定扶贫标准下贫困人口全部脱贫。教育、健康、文化、养老、安全、住房等公共服务体系更加健全，基本公共服务均等化和社会保障水平明显提高。社会主义核心价值观深入人心，闽都文化和城市精神传承发扬，市民素质、法治水平和社会文明程度显著提高，省会城市保持和谐稳定。

　　——人居环境更加优美。中心城市功能加快完善，老城区提升和新城区拓展协调推进，现代化国际城市建设取得实质性进展。新型城镇化加快推进，城乡面貌得到新改善，城乡统筹发展进入新阶段。交通拥堵、内河污染、城市内涝等突出问题有效解决，城市绿化亮化水平明显提高，生态环境质量保持全国领先，省会城市的个性、内涵、魅力不断彰显。

　　各位代表，省第十次党代会作出了“再上新台阶、建设新福建”的战略部署，市第十一次党代会发出了“马上就办、真抓实干，为建设‘机制活、产业优、百姓富、生态美’的新福州而努力奋斗”的号召。当好带动全省经济社会发展的排头兵，是省委、省政府对福州的殷切期望。2017年，我们要紧紧围绕建设新福州、当好排头兵这一目标，坚持稳中求进工作总基调，适应把握引领经济发展新常态，以提高发展质量和效益为中心，以供给侧结构性改革为主线，适度扩大总需求，扎实开展“攻坚2017”行动，狠抓各项工作落实，推动新一届政府工作开好局、起好步。今年经济社会发展主要预期目标是：地区生产总值增长9%，力争更快更好些，地方一般公共预算收入同口径增长8%，固定资产投资增长12%，出口总额稳中有升，实际利用外资增长7%，社会消费品零售总额增长11.5%，居民消费价格总水平涨幅控制在3%左右，城镇居民人均可支配收入增长8%，农村居民人均可支配收入增长8.5%，城镇登记失业率控制在3.5%以内，确保完成省下达的节能减排降碳任务。为实现上述目标，我们要抓好以下四个方面工作：

　　（一）聚焦“机制活”，全面增强发展动力活力。

　　深化供给侧结构性改革。全面落实“三去一降一补”工作任务，努力实现供求关系新的动态平衡。加强产业政策引导，支持钢铁、船舶、陶瓷等行业开展产能整合、兼并重组。坚持“房子是用来住的、不是用来炒的”的定位，严格限制投资投机性购房，合理增加土地供应，促进房地产市场平稳健康发展。强化金融风险防控，优化企业资产负债结构，降低杠杆率和融资成本，提升金融服务实体经济和创新驱动发展的水平。加大减税、降费、降低要素成本的工作力度，降低各类交易成本特别是制度性交易成本。扩大有效投资，加强基础设施、产业发展、社会事业和民生保障等领域补短板，推进投资工程包建设，实施省级重点项目192项、年度计划投资480亿元，市级重点项目700项、年度计划投资2500亿元。

　　加快福州新区建设。完成《福州新区总体规划》及各项专项规划编制。积极承接省政府下放的121项省级审批权限，研究制定新区行政许可事项目录清单。发挥新区投资发展基金的作用，设立福州华侨基金。抓好神华港储电一体化、恒申合纤10万吨差别化纤维等重大产业项目建设，推进104国道连江至晋安段改线工程、长乐前塘至福清庄前高速公路等一批重大交通设施建设，启动新区国际学校、三甲医院等公共服务项目建设，年度计划投资1800亿元。

　　推进自贸试验区建设。完成福州片区实施方案第二阶段试验任务和省里新增试验任务，推出30项以上可复制、可推广的创新举措。推动中航无人机等项目落地。争取普资租赁资产交易中心、中海债权平台中心获批，在两岸人民币跨境使用等方面先行先试。加强与国机集团、庞大集团等公司对接，力争江阴整车进口突破1.5万辆。推进中国—东盟海产品交易所在越南等国家设立分中心。

　　拓展重点领域改革。全力推进市属国企改革，理顺管理体制机制，推进经营性国有资产集中统一监管，完善国有企业负责人绩效考核与薪酬确定机制，开展国有控股混合所有制企业员工持股试点，实现市属国企主板上市零的突破。深化投融资体制改革，推广PPP等融资模式，加快组建金控集团，强化海峡银行经营管理。完善“五证合一、一照一码”等商事制度改革，在自贸试验区福州片区先行实施“证照分离”改革。健全完善生态补偿和资源有偿使用制度，建立碳排放权交易市场。继续做好农村土地承包经营权、宅基地及集体建设用地确权登记颁证工作，加强土地流转管理服务，完成闽侯国家级农村集体资产股份权能改革试点工作。

　　扩大对外开放。持续开展“抓招商、促发展”专项行动，推动宝武钢铁集团不锈钢400系生产线、旭友偏光片等重大项目落地。重点支持华映光电、德艺文化创意集团等一批具有自主知识产权、自主品牌出口企业加快发展，促进外贸出口转型升级。办好第十九届海交会、第四届丝绸之路国际电影节、第三届海上丝绸之路国际旅游节等活动，加强怀安窑址等文化遗址保护，推进“海上丝绸之路：福州史迹”申报世界文化遗产，加快筹建福州海上丝绸之路博物馆。继续实施“回归工程”，吸引更多榕籍乡亲回乡投资创业。充分发挥海峡青年交流体验营地的作用，扩大海峡青年节影响力，新建3—5个台湾青年创业就业基地，加快台商投资区基础设施建设。深化国际友城合作，做好榕籍华侨华人新生代工作，加强与港澳在金融、物流、文化创意等领域的合作。做好泛珠三角、闽浙赣皖、闽东北区域协作，力争实现闽东北经济协作区内通信一体化。继续抓好援藏援疆援宁工作，加强与定西的扶贫协作以及与渭南、延安等对口合作。

　　打造最优投资软环境。推进服务企业常态化，建立市领导跟踪服务100家大企业工作机制、重点企业季度座谈服务机制，及时解决企业各类问题。推进审批服务便捷化，力争40%的行政审批、70%的公共服务事项实现以小时为单位的服务。推进政务服务智慧化，拓展行政审批全流程电子证照应用，加快“企业服务网上平台”“惠企政策发布平台”建设。加强与重点城市环境质量的对比，加大福州优美生态环境宣传力度，促进招商引资。

　　（二）聚焦“产业优”，持续做大做强省会经济。

　　构建创新型省会城市。按照“一区多园”模式，加快推进国家自主创新示范区福州片区建设。加快中科院海西研究院三期、国家知识产权专利审查协作分中心、力普国家级海洋科研基地等创新平台建设。落实扶持“双创”7条措施，抓好福州新区国家首批双创示范基地建设，新增科技孵化器5家以上、众创空间10家以上。落实鼓励引进高层次人才7条措施，打造吸引人才的“洼地”。落实促进现代职业教育发展5条措施，加强技能型人才培养。

　　壮大都市现代农业。认真实施“粮安工程”，确保粮食种植面积稳定在150万亩、总产量达52万吨。加快发展特色优势农业和品牌农业，积极发展设施农业、生态农业、休闲观光农业和林下经济，新扩建设施农业重点项目44个。推进农业标准化生产基地建设，完善农资监管和农产品质量安全追溯监管信息平台。推动闽清澳洲肉牛羊产业基地项目落地。推广福清国家现代农业示范区试点经验。继续推动连江、长乐、罗源、闽清等省、市级农民创业园建设，建立农业科技试验示范基地20个，培训新型职业农民1000名。大力发展远洋捕捞，鼓励开展境外养殖，建设一批远洋渔业产业园、综合性远洋渔业境外基地。扎实开展第三次全国农业普查工作。

　　提升传统制造业。大力振兴实体经济，落实扶持企业技改、研发、上市、创建品牌16条措施，实施重点技改项目114个，完成技改投资860亿元，着力培育一批上市企业和国内、国际品牌。推进实施质量强市战略，创建全国质量强市示范城市。纺织化纤产业重点加快山力化纤、唐源合纤等项目建设，实现产值2800亿元。轻工食品产业重点加快元洪国际食品产业园等项目建设，实现产值1900亿元。机械制造产业重点加快东南汽车、马尾造船、上润精密等龙头企业发展，实现产值1700亿元。电子信息产业重点加快京东方8.5代面板、东旭光电玻璃基板等项目建设，实现产值1300亿元。冶金建材产业重点加快中铝集团铝精深加工、博那德低碳建筑生产一体化等项目建设，实现产值1200亿元。石油化工产业重点加快申远聚酰胺、中景石化产业园等项目建设，实现产值300亿元。

　　培育新兴产业。落实加快大数据产业发展3条措施，依托中国东南大数据产业园，推进国家健康医疗大数据中心、海上丝绸之路海洋大数据中心、国家信息中心360安全大数据中心、浪潮集团（东南）运营总部等项目落地，做好国家级互联网骨干直联点建设，大力发展大数据产业。落实加快物联网产业发展3条措施，促进窄带物联网开放实验室落地，推动物联网在水务等行业的应用，加快马尾物联网产业园区建设，大力发展物联网产业。加快三峡海上风电装备产业园、永泰抽水蓄能电站等项目建设，大力发展新能源产业。加快中科院海西研究院新材料创新研制与支撑平台、阿石创新型靶材等项目建设，大力发展新材料产业。

　　发展现代服务业。全力推进全国“十三五”服务业综合改革试点。落实加快总部经济发展的扶持政策，力争新引进市级总部企业20家以上。加快兴业资产管理公司落地，推动福建金融结算中心、福建茶业联合交易中心等项目筹建，吸引设立一批股权投资基金。加快国家电子商务示范城市建设，推进大龙网、香港中睿等电子商务项目落地。持续推进大东街口商圈改造提升，培育壮大东二环泰禾广场等新兴商圈。进一步打响“闽都文化、温泉养生、滨江滨海、清新生态”等旅游品牌，推动温泉资源综合开发利用，做旺“两马”旅游航线，加快环马祖澳旅游发展，推进琅岐国际生态旅游岛等四大滨海旅游度假区建设。办好第72届中国教育装备展示会、2017中国饲料工业展览会等大型展会。

　　建设“海上福州”。认真实施“海上福州”建设工作方案，以发展港口经济为着力点，集中力量打造福州国际深水大港。加快江阴港区6—9号泊位、罗源湾港区1—3号和14号泊位等项目建设，完善港口配套设施。加快江阴工业区化工新材料专区、罗源湾南岸可门临港产业基地、罗源湾北岸冶金建材产业基地等园区建设，大力发展临港工业。加快利嘉国际物流园、福建捷威仓储等项目建设，大力发展临港物流。加快蓝色经济产业园、临空经济区等项目建设，大力发展海洋特色产业。争取罗源湾港区扩大开放通过国家级验收。

　　打造专业特色园区。优化管理体制、运行机制，理顺园区与辖区关系，不断激发园区活力。全面落实《推进园区特色发展鼓励异地招商试行办法》，明确23家重点产业园区招商方向，引导企业向同类产业或配套产业相对集中的园区集聚，推进园区差异化、特色化、专业化发展。推进水、电、道路等生产配套和学校、商贸、文化娱乐、职工公寓等生活配套设施建设，提升园区公共服务能力，增强园区吸引力。

　　（三）聚焦“百姓富”，切实推进社会民生各项事业。

　　坚决打赢脱贫攻坚战。落实脱贫攻坚责任制，实施“造福工程”搬迁脱贫、发展教育脱贫、医疗救助脱贫、低保兜底脱贫，加大老少边贫岛地区扶贫力度，确保全市农村建档立卡扶贫对象全部脱贫。积极引导民营企业、社会组织和个人参与扶贫，探索制定稳定脱贫长效机制。加强县（市)区对口协作，实现永泰省级扶贫开发工作重点县“摘帽”。

　　提高城乡居民收入。争取城镇新增就业12.4万人，转移农业富余劳动力3.8万人，城镇失业人员再就业8500人，实施职业技能培训6万人次。全面实施创业福州助推行动计划，统筹推进高校毕业生、就业困难人员和退役军人等重点群体就业创业，加快中国福州留学人员创业园建设。健全企业职工工资决定和正常增长机制、劳动关系协调机制，完善最低工资、工资支付保障和企业工资集体协商制度，构建和谐劳动关系。

　　促进教育全面发展。实施第三期学前教育行动计划，新改扩建中小学15所、公办幼儿园13所，新增中小学学位8700个、公办幼儿园学位3600个。继续实施“校安工程”，改造校舍面积5万平方米，全面改善义务教育薄弱校办学条件。强化师德建设，加强骨干教师、学科带头人和优秀校长的培养，完善教师交流制度。大力开展素质教育，争取实现青少年校外活动中心县（市）区全覆盖。加快现代职业教育发展，全力对接省职业教育园区项目，培育2个市级以上中等职业教育公共实训基地。支持市属高等院校建设，完善福州地区大学新校区基础设施配套。支持民办教育发展。抓好特殊教育、老年教育、继续教育、终身教育。

　　保障群众健康。加快建设市妇幼保健院新院、市一医院外科楼等重点项目。进一步推进公立医院综合改革，提高基层医疗服务能力，完善分级诊疗制度，鼓励医师多点执业。加大全科医生培养力度，实施名医、名科工程。支持中医药事业发展，建设福州儿科名中医馆。完善计划生育服务管理。持续开展爱国卫生运动。加强卫生应急队伍装备建设，提升疾病预防控制和突发公共卫生事件应急处置能力。强化医德医风建设，构建和谐医患关系。积极备战第16届省运会，继续办好环福州·永泰国际公路自行车赛、中国羽毛球公开赛、中华龙舟大赛等大型体育赛事。

　　推进文化惠民。建成市图书馆新馆，规划建设船政文化博物馆新馆、市少儿图书馆新馆。全面完成基层公共文化设施达标提升工程，推进国家公共文化服务体系示范区创建。加强历史文化名城和文化遗产保护，持续推进三坊七巷、上下杭、冶山、烟台山等历史文化街区（风貌区）保护修复。深入挖掘闽都文化，办好船政文化、漆文化等文化交流活动。鼓励发展文化产业，加快海峡非物质文化遗产生态园等文化产业园区建设。大力支持文艺创作，促进哲学社会科学、新闻出版、广播影视、文学艺术等事业繁荣发展。

　　完善社会保障体系。实施全民参保登记计划，进一步提高城乡低保和城乡居民医保财政补助标准，探索建立城乡居民医保个人筹资长效增长机制。加快保障性安居工程建设，新开工建设1.3万套，基本建成1万套。积极应对人口老龄化，健全城乡养老服务体系，继续推进养老服务场所建设，鼓励社会力量参与养老服务。加快市、县两级社会福利中心以及农村幸福院建设，积极发展社会救助、慈善和残疾人事业。坚持“五个最严”的要求，创建国家食品安全示范城市，加强从“农田到餐桌”全过程监管，让群众吃得放心、安心。加强药品安全监管，保障群众用药安全。

　　深化精神文明建设。大力开展以中国梦和社会主义核心价值观为引领的精神文明建设，加强社会公德、职业道德、家庭美德和个人品德教育。积极推进群众性精神文明创建，深化文明城市、文明村镇、文明单位（行业）、文明家庭、文明校园创建活动。强化未成年人思想道德建设，鼓励发展志愿服务。深入实施“有福之州·文明同行”工作方案，全力创建第五届全国文明城市。加快信用福州建设，开展自然人信用信息征集和查询工作，初步建成市、县两级公共信用信息共享平台，加强守信联合激励和失信联合惩戒，促进形成崇尚诚信、践行诚信的社会风尚。

　　维护社会安定稳定。积极应用互联网技术，新建1万路高清视频监控系统，完善社会治安立体化防控体系，推进更高水平的平安福州建设。切实抓好“七五”普法，推进公共法律服务体系建设。健全重大决策社会稳定风险评估机制和突发事件应急处置机制，加大社会矛盾纠纷调处力度。强化安全生产监管和隐患排查整治，坚决防范重特大事故发生。推动社会组织多元健康发展，支持工会、共青团、妇联等人民团体发挥更大作用。加强民族工作，依法管理宗教事务。扎实做好防震减灾、科普、气象、保密等工作。巩固提升双拥模范城创建成果，全力支持驻榕部队和武警部队调整改革，加强国防教育、国防动员和海防、人防、反走私等工作，推进军民融合发展取得新成效。

　　（四）聚焦“生态美”，加快构建和谐宜居城市。

　　加快建设滨海新城。坚持规划引领，着力将滨海新城打造成为现代化国际新城、福州新区核心区、产城融合发展的宜居宜业智慧城市。坚持基础设施先行，加速福平铁路福州至松下段等项目建设，加快推进福平铁路莲花山延伸至机场城际铁路的前期工作，开展新一轮轨道交通线网规划修编及第三轮建设规划编制报批，动建文松快速路、东部快速通道二期工程等项目。坚持产城融合，依托空港、海港、信息港等现有载体与产业基础，把滨海新城打造成为全国重要的区域大数据中心。

　　改善城市宜居环境。落实城区水系治理工作方案，完成晋安河重点段疏浚、清障和生态驳岸改造，完成井店湖等“四湖一园”建设；建设雨水管网50公里、污水管网50公里，实现四城区42条水系的水体黑臭基本消除，20条以上的河道水质达标；完成沿河16个旧屋区拆迁，新增水系周边绿化92万平方米，基本杜绝内河沿岸污水超排和污水偷排。落实缓解城区交通拥堵工作方案，加快打通二环五四路口、尤溪洲北桥头等重要堵点，完成125个整治项目，实现出行环境显著改善。推进南北火车站、长乐机场整治，严厉打击机场非法营运，完成127个南北火车站整治项目。大力推进“全民动员、绿化福州”专项行动，完成359个绿化项目，建成7个榕树、茉莉花主题公园，改造提升39条林荫路，建成12条休闲步道，通过国家森林城市考核验收。提升城市亮化水平，实施闽江两岸等重要节点夜景灯光改造。加快新型智慧城市标杆市建设步伐，提高城市管理精细化水平。继续抓好鹤林片区等海绵城市建设试点，积极申报国家第三批综合管廊建设试点城市。

　　增强城市承载功能。积极在海峡西岸城市群建设中发挥中心引领作用。启动新一轮城市总体规划修编，编制完成福州市空间发展战略规划。推进城市“双修”工作。制定出台优化综合交通体系建设方案，推进福厦客运专线、洪山大桥等项目建设。全面加快地铁1号线延伸段、2号线、6号线建设，动建5号线，争取动建4号线。新改扩建福马路等47条市政道路，打通湖东东路、义北路北段等断头路，推进南台大道南段等“接二连三”工程建设。大力实施公交优先战略，新增更新公交车300辆，优化公交线路20条，新投放便民自行车3000辆。新增公共停车泊位4000个。加快推进超山变电站、洋洽变电站等45个重点输变电项目建设，优化电力设施布局。

　　统筹城乡协调发展。推动福清市国家新型城镇化综合试点，提升江阴、青口省级“小城市”试点建设水平，加快永泰嵩口、长乐东湖VR小镇等国家级、省级特色小镇建设，争取新建1—2个省级以上特色小镇。继续实施新农村“幸福家园工程”，建设425个示范村。持续开展以垃圾、污水为重点的农村环境综合治理，新建100个美丽乡村、12个美丽乡村示范村、4条美丽乡村景观带。创建9个乡村旅游精品示范点、25个乡村旅游特色村和1个休闲集镇。加强古村落、古建筑的保护修复，继续推进历史文化名镇、名村建设。

　　推进生态文明建设。全面落实《国家生态文明试验区（福建）实施方案》和我市贯彻意见，完成15项改革任务，抓好长乐、闽清、永泰生态文明体制改革试点。严格落实生态红线保护制度，实施生态环境分区分级管控，加快永泰国家重点生态功能区示范试点建设。继续实施“大气十条”及提升环境空气质量行动计划，保持空气质量位居全国74个重点城市前列，实现“保八争四”目标。落实河长制，加强小流域治理、饮用水水源地保护。加大土壤污染防治力度，启动建设餐厨垃圾和危险废物处理场，依法严厉查处非法排放有毒有害污染物等违法行为。深入开展节能减排，实施30项节能技术改造项目，执行县（市）区污染物减排刚性约束。大力发展绿色循环经济，积极倡导垃圾分类、绿色消费、低碳出行的生活方式。

　　三、马上就办、真抓实干，建设为民务实廉洁的服务型政府

　　各位代表，福州新一轮的发展，既有机遇，更有挑战。我们必须进一步振奋精神，转变职能，改进方法，提速增效，不断开创政府工作新局面。

　　坚定政治方向。牢固树立“四个意识”，严明政治纪律和政治规矩，自觉向党中央看齐，向习近平总书记看齐，向党的理论和路线方针政策看齐，向党中央的决策部署看齐，始终在思想上政治上行动上同以习近平同志为核心的党中央保持高度一致。坚决维护市委在推动福州发展中总揽全局、协调各方的领导作用，以实际行动确保党的政令畅通、决策落地生根。

　　严格依法行政。加快法治政府建设，严格按照法定权限和程序行使权力、履行职责。健全完善政府法律顾问制度，做好规范性文件备案和审查。主动接受市人大及其常委会的法律监督、工作监督和市政协的民主监督，加强与各民主党派、工商联和各人民团体、社会各界人士的沟通联系，进一步提高办理市人大代表建议、市政协提案的落实率和满意率。完善政府议事规则和决策程序，全面推进政务公开，主动接受舆论监督和群众监督。

　　提升行政效能。秉持系统、综合、整体的工作理念，运用针对问题提出措施、对照措施生成项目、盯紧项目抓好督查的工作方法，更好更快促进工作任务全面落实。坚持全体动员、组团攻坚、集中发力，积极破解阻碍发展的瓶颈制约。深化机关效能建设，持续整治“为官不为”“庸懒散拖”等不良作风。

　　强化责任担当。加强一线考察干部，坚持在重大项目建设、招商引资、灾后重建等急难险重任务和日常工作一线培养干部、识别干部、使用干部。深入开展“四个万家”等活动，引导广大干部深入基层、服务群众、促进发展。倡导敢想敢试、敢闯敢为，让想干事的人有机会，能干事的人有舞台，敢干事的人有发展，进一步提振干事创业的“精气神”。

　　改进工作作风。大力弘扬“马上就办、真抓实干”精神，态度再坚决一点、行动再快一点，以敢于担当的使命感、时不我待的紧迫感、奋起赶超的危机感，雷厉风行，紧抓快办，一抓到底，全力推动落实。坚持高标准、严要求，不甘平庸，自加压力，勇于争先，敢争一流。

　　加强廉洁建设。严格执行党内政治生活若干准则、党内监督条例等党内法规，持之以恒落实中央八项规定精神。强化行政监察，积极推进审计监督全覆盖，加强对行政权力运行的制约和监督。健全完善惩治和预防腐败体系，增强拒腐防变能力。

　　各位代表，建设新福州、当好排头兵，责任重大、任务艰巨。光荣的使命，鼓舞我们砥砺奋进；人民的期盼，鞭策我们勇往直前。让我们更加紧密地团结在以习近平同志为核心的党中央周围，在市委的坚强领导下，凝心聚力，开拓进取，全力拼搏，加快建设“机制活、产业优、百姓富、生态美”的新福州，以优异成绩迎接中国共产党第十九次全国代表大会的胜利召开！
